# Health economics assessment of statin therapy initiation thresholds for atherosclerosis prevention in China: a cost-effectiveness analysis

**DOI:** 10.1186/s12939-025-02391-9

**Published:** 2025-01-24

**Authors:** Tianyu Feng, Xiaolin Zhang, Jiaying Xu, Shang Gao, Xihe Yu

**Affiliations:** 1 School of Public Health, Chongqin Medical University, Chongqing, 400016 Chongqing China; 2 School of Public Health, Jilin University, Changchun, 130021 China

**Keywords:** Statins, Cost-effectiveness analysis, CHARLS, Risk threshold

## Abstract

**Background:**

Recent updates to the Chinese guidelines for dyslipidemia management have reduced the 10-year risk threshold for starting statins in the primary prevention of atherosclerotic heart disease. This study aims to evaluate the potential negative effects of different statin initiation thresholds on diabetes risk in the Chinese population, while also analyzing their health economic implications.

**Methods:**

I We developed a microsimulation model based on event probabilities to assess the cost-effectiveness of statin therapy. The model utilized the China-PAR prediction tool for ASCVD risk and incorporated data from a nationally representative survey and published meta-analyses of middle-aged and elderly Chinese populations. Four strategies were evaluated: a 7.5% 10-year risk threshold, the current guideline strategy, and a 15% threshold. For each strategy, we calculated the incremental cost per quality-adjusted life year (QALY) to gain insights into the economic impact of each approach.

**Result:**

The incremental cost per QALY for the 10% 10-year risk threshold strategy, compared to the untreated, was $52,218.75. The incremental cost per QALY for the guideline strategy, compared to the 7.5% 10-year risk threshold strategy, was $464,614.36. These results were robust in most sensitivity analyses.

**Conclusion:**

Maintaining the recommended thresholds outlined in the current guidelines for the management of dyslipidemia may represent a cost-effective option for China at present. Variations in statin prices and the risk of statin-induced diabetes have significant impacts on the cost-effectiveness outcomes.

**Supplementary Information:**

The online version contains supplementary material available at 10.1186/s12939-025-02391-9.

## Introduction

In the past three decades, China’s disease profile has shifted significantly, with cardiovascular diseases now overtaking infectious diseases as the leading cause of death [1]. Over 40% of deaths in China are attributed to cardiovascular conditions, and dyslipidemia is a key risk factor for atherosclerotic cardiovascular disease (ASCVD) [2, 3]. To mitigate the risk of major cardiovascular events, clinical guidelines worldwide recommend statin therapy for managing dyslipidemia [4–6]. Most guidelines use a combination of risk factor-based and absolute risk-based criteria to determine when statin therapy should be initiated, though the recommended thresholds vary. For instance, China’s 2016 guidelines suggest starting statins when the 10-year ASCVD risk is 10% [4], while Scotland sets the threshold at 20% [7]. Some countries have adopted lower thresholds, such as the American Heart Association (AHA) in its 2018 guidelines and the UK’s National Institute for Health and Care Excellence (NICE) in its 2023 update, both recommending statin therapy when the 10-year ASCVD risk reaches 7.5% [5, 6]. An Australian study found that a 15% 10-year risk threshold was more cost-effective than thresholds of 5% or 10% for patients with coronary heart disease (CHD) and stroke [8]. Considering economic value is crucial when developing statin initiation guidelines, particularly in developing countries like China.

Chinese guidelines currently recommend starting statin therapy when a patient’s 10-year ASCVD risk exceeds 10%, but this threshold remains a point of debate in academic circles [4, 9]. Economic factors are crucial in setting this threshold. A lower threshold could increase treatment costs for less at-risk groups without significant health benefits, while a higher threshold may lead to undertreatment of patients who could benefit from statins. Despite their effectiveness, statins carry risks, notably the increased likelihood of developing type 2 diabetes with long-term use (statin-induced diabetes). In 2012, the U.S. Food and Drug Administration (FDA) issued a warning about the potential for statins to raise blood sugar and hemoglobin levels [10]. Studies have shown that extended statin use significantly heightens the risk of type 2 diabetes [11–13]. Given China’s status as the country with the highest diabetes burden [14], it is vital to explore how different statin initiation thresholds impact diabetes risk, alongside analyzing the cost-effectiveness of statins in preventing ASCVD. This study aims to guide clinicians and policymakers in developing safe, effective, and economically viable dyslipidemia management strategies. Each country must determine its own cost-effective statin initiation threshold based on its unique population, healthcare infrastructure, and economic conditions.

## Methods

### Model structure

This study utilized a microsimulation model to predict lifetime outcomes and costs related to ASCVD for individuals aged 40 to 89 [15]. The model operates on a modified Markov state model, where individuals transition between health states based on specific probabilities. All parameters of the simulated subjects in this study, including age, gender, physiological indicators, medical history, and treatment status, were derived from the survey results of the 2015 China Health and Retirement Longitudinal Study (CHARLS), which encompassed a total of 20,967 respondents. After excluding participants who lacked complete blood data, body measurements, sociodemographic data, as well as those who were younger than 45 years old, a total of 12,398 participants were ultimately included in the analysis. The model caps at age 89 due to the limited number of individuals in the Chinese population aged 90 and above [16].

**Fig. 1:**
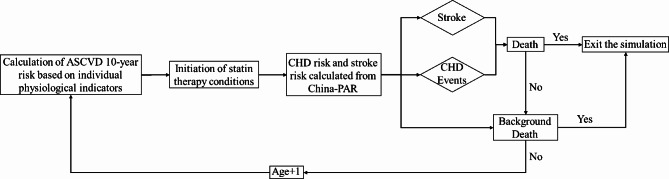
Structure of the microsimulation model. CHD events include all fatal coronary events and non-fatal AMI

The model operates in one-year cycles, with cost and health utility values updated at the end of each cycle. If an individual does not die or reach the maximum modeled age, the simulation continues into the next cycle. When an individual dies or reaches the age limit for the simulation, the model terminates, and all costs, health outcomes, and recorded events are summarized for the entire simulation period. The structure of the model is illustrated in Fig. 1, and it was implemented using Treeage Pro2019 software.

### Simulation population

At the start of the simulation, the model included 10,000 individuals with no history of ASCVD. ASCVD-related risk factor parameters were sourced from the 2015 CHARLS, with all other model inputs detailed in the supplementary material (supplementary material Tables S1 to S4) [17]. To simulate changes in ASCVD risk factors (including HDL-C, LDL-C, TC, SBP, and WC) over time, cross-sectional linear regression was applied to CHARLS data to determine the relationship between these risk factors and age, as part of the China-PAR risk function (supplementary material Tables S5 and S6) [18]. Data on diabetes prevalence and the age-specific increased risk of statin-induced diabetes were gathered from published studies [19].

### Risk of ASCVD events

This study employed a total risk equation from China-PAR to estimate the 10-year composite risk of ASCVD events [9, 20]. Both the 2016 revised Chinese guidelines for dyslipidemia management and the cardiovascular disease prevention guidelines recommend China-PAR as a reliable ASCVD risk assessment tool [4]. The China-PAR model (Prediction for Atherosclerotic Cardiovascular Disease Risk in China) uses physiological parameters—such as HDL-C, LDL-C, total cholesterol (TC), systolic blood pressure (SBP), and waist circumference (WC)—along with medical history and treatment status to calculate the ASCVD risk for each cycle. It has been widely validated and used in clinical practice across China. The China-PAR model predicts fatal coronary heart disease (CHD) events, including those caused by acute myocardial infarction (AMI) or other coronary deaths, as well as non-fatal AMI and fatal/non-fatal stroke. The 10-year risk was converted into a 1-year risk using the DEALE method [18]. The composite 10-year probability of fatal AMI includes 29.46% for CHD and 70.54% for stroke, based on their relative shares of total events [21]. Patients who survived a CHD or stroke event without recurrence experienced more than double the background mortality rate during follow-up [22]. The probability of diabetes events also comes from the 2015 CHARLS survey supplement (supplementary material Table S4). For individuals on statin therapy, the probability of diabetes is adjusted according to the relative risk of statin-induced diabetes [23].

### Treatment strategies

The treatment initiation strategy recommended by the guidelines is shown in electronic supplementary material Figure. S1. This study examines statin regimens based on the 2023 edition of the Chinese guidelines for lipid management, particularly focusing on the 10-year ASCVD risk threshold. The China-PAR risk equation was employed to estimate patients’ 10-year risk of developing ASCVD [24]. The guidelines advise the use of moderate-intensity statins for primary prevention [4], while high-intensity statin therapy was excluded from analysis, as it is recommended to be used with caution in the Chinese population. Previous studies assumed that 40% of patients would discontinue statin therapy after one year, and that all patients should ideally adhere to long-term treatment [25, 26].

Given that diabetes notably increases the risk of ASCVD and statins can slightly raise the likelihood of developing type 2 diabetes [18, 27], this study examines the impact of statin-induced diabetes on the overall ASCVD risk [28, 29]. It was assumed that patients who developed diabetes due to statin use had well-controlled blood glucose and no other comorbid conditions. The simulation model only accounted for the effects of statin-induced diabetes on quality of life and healthcare costs.

We evaluated 10-year risk thresholds of 7.5%, 10%, and 15% to determine optimal treatment initiation points. The simulated regimens aligned with the strategies outlined in the guidelines, utilizing the China-PAR risk function for treatment initiation. Additionally, simulations without treatment were conducted.

### Cost and health utility

To ensure the accuracy and credibility of the parameters, the cost and health utility value parameters employed in the microsimulation are rigorously derived from highly reliable and authoritative research findings. The specific details and detailed sources of each parameter are presented in Table 1.

This study adopts the perspective of the Chinese healthcare system, focusing exclusively on direct medical costs related to ASCVD, including expenses for statins, inpatient care, and outpatient services. Drug costs were determined based on the national negotiated prices from 2020. We employed a methodology similar to that of a prior study, calculating annual drug expenses by averaging the lowest winning prices of statins listed in local government procurement catalogs from various regions of China in the first quarter of 2020. Assuming that individuals recovering from CHD or stroke require one outpatient visit per year for the rest of their lives, we adjusted the costs to 2022 using the China Healthcare Component Consumer Price Index.

**Table 1:** Parameters used in the model

Parameter	Value		Distribution	Standard error	References
Acute disutility					[30]
Coronary heart disease	0.439		Beta	0.018	
Stroke	0.92		Beta	0.04	
Post-event long-term disutility					[30,31]
Acute myocardial infarction	0.107		Beta	0.019	
Stroke	0.266		Beta	0.02	
Treatment effect on CHD (OR)	0.7		Log-normal	0.072	[24]
Treatment effect on stroke (OR)	0.81		Log-normal	0.069	[24]
Risk of statin-induced diabetes (OR)	1.21		Beta		[23]
Annual statin treatment costs	1,149.75		Gamma		
Costs (2022 CN¥)	AMI	Stroke			
Hospitalization costs	22,611	13,983	Gamma		[32]
First-year long-term costs	5,255.85	2652	Gamma		[33]
Office visit costs in subsequent years	585.87	565	Gamma		[34]
CHD mortality by age group	Men	Women			[33]
34–44	0.12	0.18	Beta		
45–54	0.21	0.23	Beta		
55–64	0.29	0.27	Beta		
65–74	0.33	0.43	Beta		
75–84	0.48	0.51	Beta		
Stroke mortality by age group	Men	Women			[33]
34–44	0.25	0.18	Beta		
45–54	0.18	0.14	Beta		
55–64	0.12	0.15	Beta		
65–74	0.2	0.2	Beta		
75–84	0.45	0.45	Beta		

Quality-adjusted life years (QALYs) were determined using age-specific utility weights along with event-related negative utility weights, which are further divided into acute and long-term negative utility for patients who survive only the acute phase (Table 1). The age-specific utility weights were obtained from the EQ-5D survey conducted among the Chinese general population (supplementary material Table S2) [35]. Acute and long-term negative utility weights were sourced from the Global Burden of Disease project and the EQ-5D survey of chronic disease patients in China [30]. For the baseline case, an annual discount rate of 3% was applied [36].

### Intervention strategy comparisons

This study assesses strategies using the incremental cost-effectiveness ratio (ICER). The cost-effectiveness ratio for each alternative is calculated, and less effective strategies are excluded. China’s gross domestic product (GDP) per capita for 2022 (CNY 85,700) serves as the threshold for cost-effectiveness, while three times this amount (CNY 257,100) is considered the minimum criterion for a strategy to be deemed cost-effective [37]. Additionally, the study utilizes cost-acceptability curves to demonstrate the likelihood that each strategy is cost-effective at various willingness-to-pay (WTP) levels.

### Sensitivity analysis

We conducted one-way and probabilistic sensitivity analyses (PSA) on the model parameters to evaluate the robustness of our results with varying parameters. The univariate sensitivity analysis concentrated on the impacts of statin effectiveness, cost, and the risk of diabetes on the optimal ASCVD treatment threshold. In the PSA, we examined overall model uncertainty through 1,000 randomized simulations.

### Model validation

The model utilized in this study was adapted from a thoroughly validated microsimulation model [15, 38]. Its internal and external validity were confirmed based on the model’s outputs [39, 40]. Internal validity was assessed through regression analysis, comparing the expected probabilities of CHD and stroke events for different age and sex groups in the untreated model against the average simulation results (supplementary material Figure S2). External validity was established by comparing the simulated incidence of ASCVD within specific age-sex groups (10-year intervals) to findings from other studies (supplementary material Table S7) [41].

## Results

### Model calibration and validation

Figure S2 in the supplementary material presents the internal validation results of the model. The coefficient of determination (R²) for the linear regression comparing the mean 1-year expected probabilities of CHD and stroke events with the corresponding simulated values for the untreated group is 0.9278, indicating strong internal validity. The simulated stroke incidence shown in Table 2 closely matches the findings from prospective studies. Currently, China’s published life expectancy per capita is 75.4 years, while the untreated model predicts a life expectancy of 74.9 years, further supporting its external validity.

### Clinical event results

An ASCVD threshold of 7.5% or higher was found to make approximately 48% of adults eligible for statin therapy. Strategies that employed higher ASCVD thresholds were associated with fewer cases of statin-induced diabetes but resulted in more CVD events. In the context of the 573 million adults aged 40 to 75 in China, lowering the ASCVD threshold from 15 to 10% was estimated to prevent an additional 1,088,716 CVD events. Furthermore, reducing the threshold from 10 to 7.5% was associated with avoiding an estimated 687,610 additional CVD events. These findings highlight the overall benefit of statins in mitigating cardiovascular events.

### Cost-effectiveness analysis

Table 2 displays the base case results of this study. The ICERs for the 15% risk threshold strategy compared to no treatment were $43,951.82 per QALY. Since the 15% risk threshold strategy is an expansion advantage strategy, it was excluded from further analysis. The ICERs for the guideline strategy compared to the untreated group were $52,218.75 per QALY. In comparison, the ICERs for the 7.5% risk threshold strategy versus the guideline strategy were $464,614.36 per QALY. Consequently, under the 3-GDP criterion, the 7.5% risk threshold strategy emerged as the most optimal among the three strategies. Conversely, under the 1-GDP criterion, the 10% risk threshold strategy was determined to be optimal.

### Sensitivity analysis

**Fig. 2:**
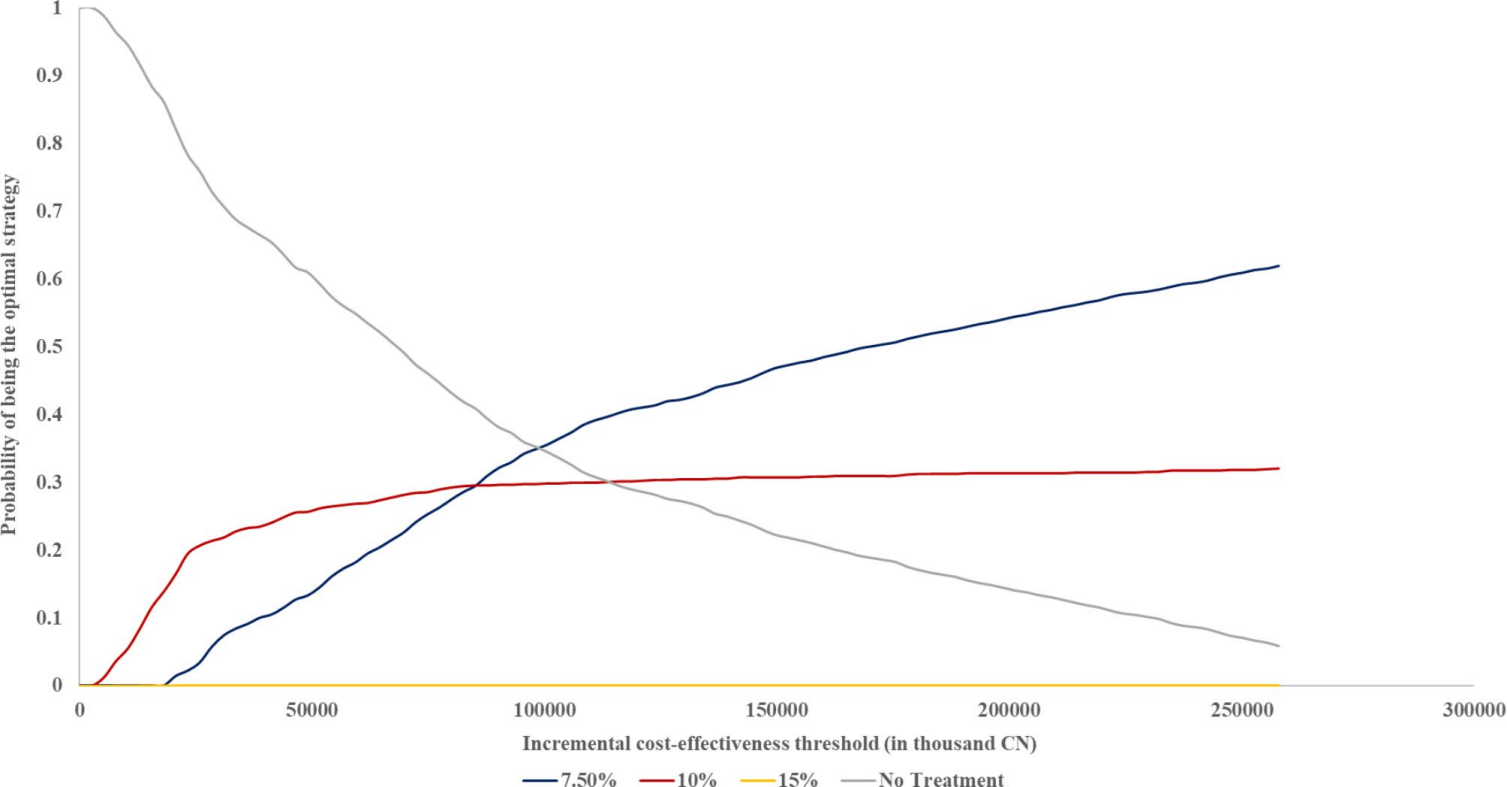
Acceptability curves were generated using probabilistic sensitivity analysis results. The horizontal coordinate of the figure represents WTP, calculated in RMB

Figure 2 illustrates the acceptability curves for the PSA results. Under the 3-GDP cost-effectiveness criterion, the guideline strategy has a 32% probability of being the most optimal, while the 7.5% risk threshold strategy has a 62% probability. When applying the 1-GDP criterion, both the guideline strategy and the 7.5% risk threshold strategy demonstrate equal cost-effectiveness, each with a 30% probability of being optimal. The cost-acceptability curve indicates that the current guideline strategy is more cost-effective than the 7.5% risk threshold strategy when the WTP is below 1-GDP.

**Fig. 3:**
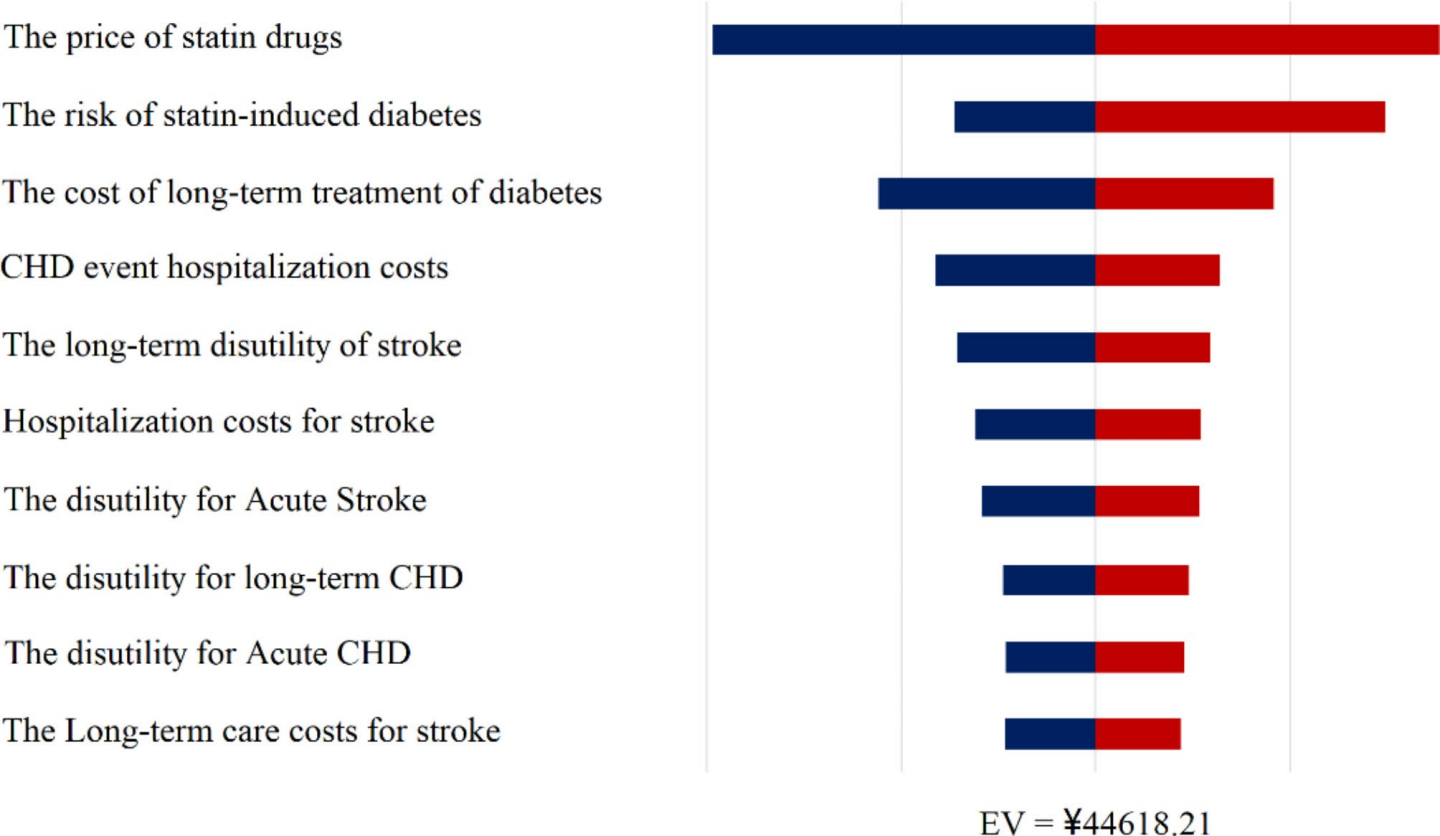
The tornado plot displays the results of the deterministic sensitivity analysis. The price of statins and the risk of statin-induced diabetes had the greatest impact on the results

Figure 3 presents the results of the one-way sensitivity analysis in a tornado plot. When all parameters are adjusted within their variability ranges, the changes in the ICER remain within an acceptable range around 1-GDP. Notably, fluctuations in statin prices and the risk of statin-induced diabetes exert the most significant influence on the cost-effectiveness outcomes.

**Table 2:** Base-case results of ICERs

ASCVD Risk Threshold, %	Adults Statin Eligible, %	Statin-Induced Diabetes Cases	CVD Events	Mean discounted QALYs	Life Expectancy	Mean discounted costs(CN¥ in 2018)	ICER
No Treatment			0.44	12.17	75.34	49360.91	
15%	39%	0.00695	0.43	12.46	75.63	62236.63	Extended dominance
10%	47%	0.00755	0.43	12.47	75.79	65128.64	52218.75
7.50%	56.00%	0.00745	0.43	12.47	75.81	66866.81	464614.36

## Discussion

The primary objective of healthcare systems is to maximize health benefits for the population while minimizing costs, especially regarding the economics of preventive therapies [42]. Statins have proven effective in preventing ASCVD, but their use is linked to a higher incidence of diabetes, posing challenges for policymakers, healthcare providers, and patients [43, 44]. Our analysis indicated that adopting a 7.5% 10-year ASCVD risk threshold for initiating statin therapy is more effective than the current Chinese dyslipidemia management guideline’s recommendation of a 10% threshold when evaluated against the 3-GDP criterion. Sensitivity analysis results showed that the sales price of statins and the preventive strategy for statin-induced diabetes significantly influenced the outcomes. Notably, a 20% reduction in statin prices would lower the ICER for the 7.5% threshold strategy to below 2-GDP. Overall, these findings suggest that the optimal treatment threshold is contingent upon the cost-effectiveness criteria applied.

For adults without cardiovascular disease, the decision to start statin therapy should be guided by evidence-based practices and individual patient preferences. When a patient’s ASCVD risk reaches the initiation threshold, they may have legitimate personal reasons for refusing statin treatment [45]. In China, financial constraints often lead patients to stop treatment; for many lower-income individuals without comprehensive health insurance, the cost can be prohibitive [46]. Our one-way sensitivity analysis revealed that statin-induced diabetes significantly affects simulation outcomes. Therefore, it is crucial to monitor the glycemic profiles of patients on statins and conduct long-term glucose assessments.

Research examining the economic aspects of statin clinical guidelines in various countries suggests that the initiation threshold for statin therapy could be lowered. Pandya et al. evaluated the cost-effectiveness of the 2013 ACC/AHA guidelines and alternative risk thresholds for preventing ASCVD events. They found that the ACC/AHA’s 10-year ASCVD risk threshold of ≥ 7.5% was cost-effective, but further reductions in this threshold (e.g., to ≥ 3% or ≥ 4%) would enhance its cost-effectiveness [15]. Similarly, Linda J et al. reviewed cardiovascular prevention guidelines in Australia and identified that the best cost-effectiveness was achieved by recommending statins for individuals with a five-year ASCVD risk exceeding 5% or a ten-year risk surpassing 10% [8]. These findings imply that more aggressive strategies for initiating statin therapy could be worthwhile. However, since these studies largely reflect the demographics, healthcare standards, and economic conditions of developed countries, their findings may not be directly applicable to developing nations like China. Therefore, statin initiation thresholds should be aligned with the country’s WTP.

Previous economic studies of statins often neglected the impact of diabetes, limiting their ability to fully capture the true effects of statins in preventing ASCVD. Additionally, the risk functions employed in these studies do not align with the current needs of frontline healthcare, as they are difficult to access and lead to analysis outcomes that may diverge from real-world situations. This gap has made it harder for prior research to offer practical, actionable guidance. In contrast, our study enhanced the model structure and adjusted the parameters to deliver valuable insights for clinical practice. For instance, Cobiac et al. utilized the average cost-effectiveness ratio rather than ICER to evaluate different absolute risk threshold strategies [8]. Similarly, Jiang et al. assessed the cost-effectiveness of varying risk thresholds through a DEM model based on a national survey [47]. However, unlike our research, their analysis did not account for the risk of statin-induced diabetes, leaving out a critical factor in ASCVD prevention. Our study addressed this significant gap.

Based on the findings of this study, we recommend that China maintain its current statin initiation threshold. Although the simulation results indicate that the cost-effectiveness level of lowering the initiation threshold for statin therapy does not differ significantly from that of the current threshold, the absence of research reports on WTP thresholds makes it difficult for us to confidently recommend a reduction in the threshold. ASCVD is a multifaceted disease, influenced by numerous factors, and statins alone may not provide a comprehensive solution. Furthermore, the cost-effectiveness analysis shows that a 7.5% 10-year ASCVD risk threshold would only become more economically viable with a higher WTP. Given that China’s current economic status is not on par with developed nations, and patient WTP remains limited, it would be prudent for policymakers to consider relaxing the threshold only after further economic development. Moreover, prematurely lowering the initiation threshold could trigger a “broken window” effect, increasing the use of other medications, such as anticoagulants, and placing additional strain on China’s healthcare system.

Studies using Markov models often have certain limitations. Firstly, this study is a pharmacoeconomic evaluation based on retrospective observational research, and the findings still require further validation through real-world studies. Furthermore, Markov cohort models rely on average probabilities to represent individual life courses, which can make it difficult to generate more realistic simulations [48]. In this study, however, we calculated each individual’s absolute ASCVD risk using demographic and clinical characteristics from the CHARLS data, allowing treatment decisions to be tailored to individual risk profiles. Second, a key limitation of Markov cohort models is their “memoryless” property, which makes it challenging to account for the influence of historical events on patient outcomes and costs [49]. Microsimulation models address this issue by tracking each individual over time, adjusting transition probabilities and cost parameters based on historical events.

In addition to the inherent limitations of simulation-based studies, the following limitations of this study should also be acknowledged. First, the ASCVD risk function employed can only estimate the 10-year combined ASCVD risk, making it impossible to separate the risks of developing AMI and stroke, which were instead simulated using a fixed probability. Second, this study concentrated on the effect of statin-induced diabetes risk on ASCVD prevention but did not investigate the health damage or economic losses caused by complications in diabetic patients. The analysis is conducted from the national payer’s perspective, so only direct medical costs were included. A complete evaluation of the economic benefits of medications requires consideration of the financial strain on the healthcare system, societal costs, opportunity costs, and issues of equity and fairness [50]. Third, earlier research has indicated that statin adherence plays a crucial role in ASCVD prevention. Due to the lack of long-term statin adherence data in China, we relied on studies from other countries, which might not accurately reflect Chinese realities. Finally, although we incorporated the risk of statin-induced diabetes, not all possible risks and benefits were accounted for, such as the potential for reduced risk of advanced/aggressive prostate cancer, lower in-hospital sepsis mortality, and the risk of statin-induced rhabdomyolysis [51–54]. Given that statin-induced diabetes is generally regarded as the most significant negative effect, the excluded factors are unlikely to have substantially influenced the outcomes of this study [47].

## Conclusions

Based on the research findings, utilizing a 10-year ASCVD risk threshold greater than 10% as the initiation point for statin therapy is cost-effective when the WTP is set at 1-GDP. Consequently, this study suggests that China should maintain the current recommended threshold outlined in its dyslipidemia management guidelines. The decision on whether to lower this recommended threshold awaits further investigation and research for a conclusive determination. Variations in the price of statins and the risk of statin-induced diabetes have significant impacts on the cost-effectiveness outcomes.

## Electronic supplementary material

Below is the link to the electronic supplementary material.

Supplementary Material 1

Supplementary Material 2

Supplementary Material 3

## Data Availability

The datasets supporting the conclusions are available from the corresponding author (Yu X, xhyu@jlu.edu.cn) on reasonable request.

## Abbreviations

AMI: Acute myocardial infarction
ASCVD: Atherosclerotic cardiovascular disease
CHD: Coronary heart disease
CHARLS: China health and retirement longitudinal study
China-PAR: Prediction for atherosclerotic cardiovascular disease risk in China
ICER: Incremental cost-benefit ratio
PSA: Probabilistic sensitivity analyses
QALY: Quality-adjusted life year
WTP: Willingness-to-pay
