# Supplementary figures and images for: Health economics assessment of statin therapy initiation thresholds for atherosclerosis prevention in China: a cost-effectiveness analysis
Source: Int J Equity Health. 2025 Jan 24;24:31. doi: 10.1186/s12939-025-02391-9 (PMC11762857; doi:10.1186/s12939-025-02391-9)

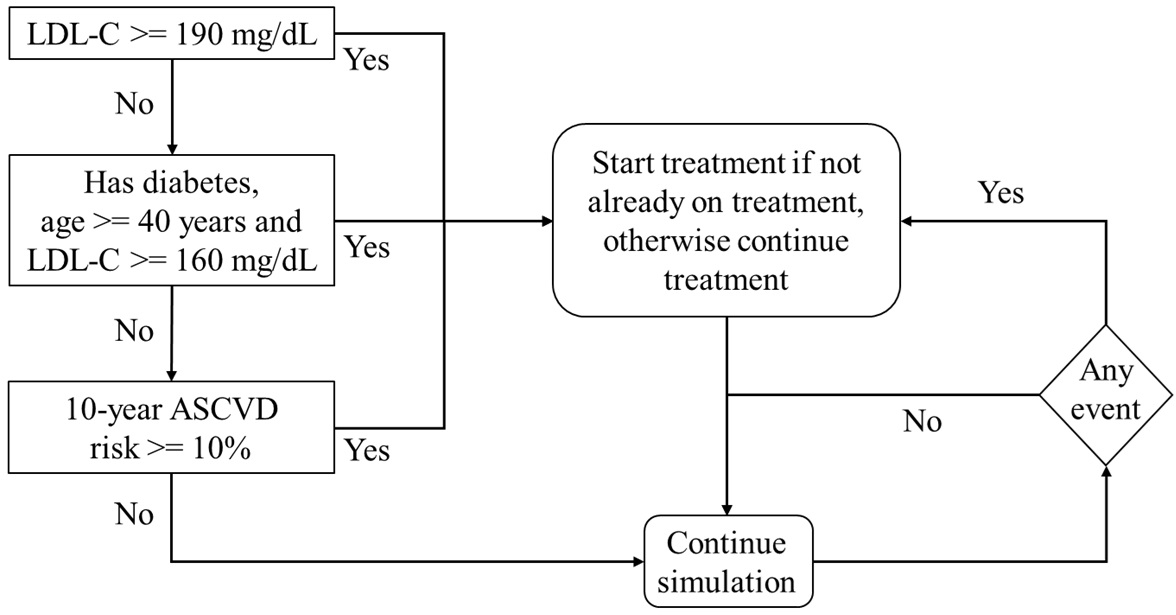

Supplement: Supplementary file 1 — Supplementary Material 1 [file 12939_2025_2391_MOESM1_ESM.jpg]

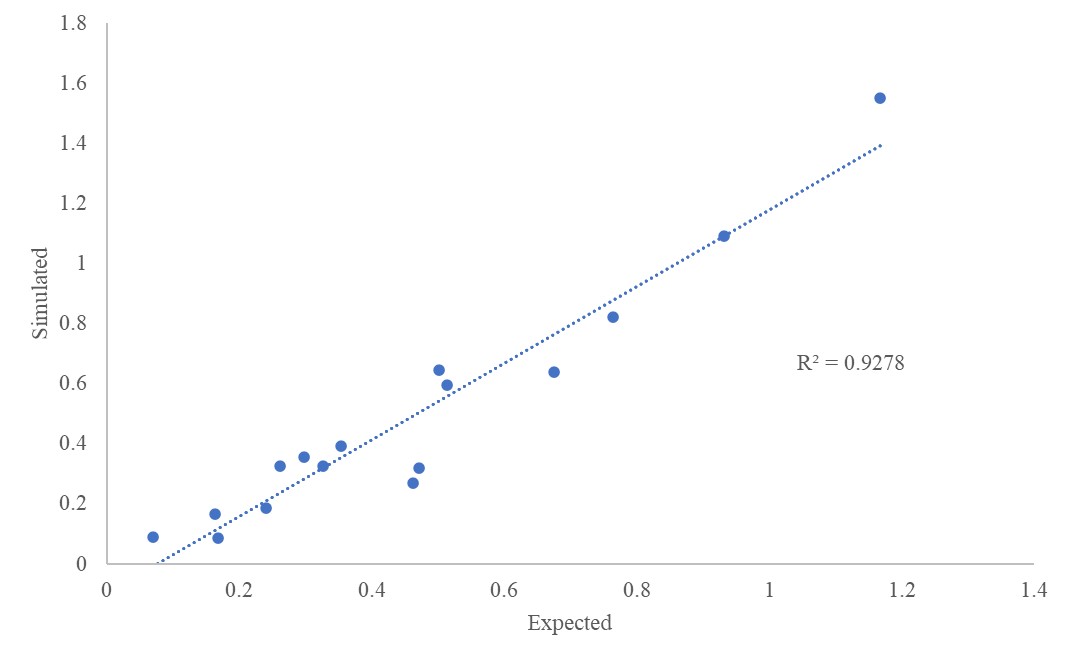

Supplement: Supplementary file 2 — Supplementary Material 2 [file 12939_2025_2391_MOESM2_ESM.jpg]
